# The Genetic Architecture of Self-Reported Psychopathy in Adults

**DOI:** 10.64898/2026.09.08.26362549

**Published:** 2026-09-10

**Authors:** Justin D. Tubbs, Travis T. Mallard, Maria Dalby, Younga H. Lee, Karmel W. Choi, Tian Ge, Niels Plath, Lene Hammer-Helmich, Julie M. Granka, 23andMe Research Team, Essi Viding, David A. Hinds, Jordan W. Smoller, Joshua W. Buckholtz

## Abstract

**Background:**

Psychopathy is a personality construct characterized by affective and interpersonal features coupled with severe antisocial behavior. While strongly associated with extreme behaviors such as violent crime, empirical work suggests that psychopathy exists as a dimensional trait in the general population. However, our understanding of the genetic structure of psychopathy remains limited, with genome-wide association studies (GWAS) having been restricted to broad measures of antisocial behavior.

**Methods:**

We perform the first large-scale GWAS of psychopathy, using total and factor scores from the Self-Report Psychopathy scale (SRP) in up to 10,558 participants enriched for mood disorder cases from the AFFECT study. LD score regression (LDSC) was used to characterize the heritability of SRP scores and estimate their genetic correlation (rG) with several externally-measured traits, using the Benjamini-Hochberg approach to perform multiple testing correction.

**Results:**

Psychopathy scores showed significant heritability, with 12-14% of phenotypic variance explained by common genetic variants. However, GWAS did not identify any genome-wide significant loci in this sample. The LDSC-estimated genetic correlation with antisocial behavior was only 0.54 (95%CI: 0.21-0.81), in line with prior twin research. Significant genetic correlations were found with a wide range of psychiatric disorders (rG’s: 0.21 - 0.72), with the highest observed for borderline personality disorder (95% CI: 0.41-0.72) and substance use disorders (95% CI: 0.43-0.63). Psychopathy was also genetically correlated with lower life-satisfaction, poorer health outcomes, worse cognitive functioning, and lower educational attainment.

**Conclusions:**

In the first large-scale GWAS of self-reported psychopathy, we found significant SNP heritability and genetic overlap with risk for psychiatric disorders, and poorer behavioral, cognitive, and physical health outcomes. These findings provide important genetic support for differentiating psychopathy from antisocial behavior. Further, they inform our understanding of how genetic factors may contribute to poor health outcomes in psychopathy, and shed insight into the comorbidity between psychopathy and other disorders.

## Full Text Availability

The license terms selected by the author(s) for this preprint version do not permit archiving in PMC. The full text is available from the preprint server.

